# Efficacy and Safety of Cyclophosphamide Treatment in Severe Juvenile Dermatomyositis Shown by Marginal Structural Modeling

**DOI:** 10.1002/art.40418

**Published:** 2018-03-25

**Authors:** Claire T. Deakin, Raquel Campanilho‐Marques, Stefania Simou, Elena Moraitis, Lucy R. Wedderburn, Eleanor Pullenayegum, Clarissa A. Pilkington, the Juvenile Dermatomyositis Research Group, Kate Armon, Joe Ellis‐Gage, Holly Roper, Vanja Briggs, Joanna Watts, Liza McCann, Ian Roberts, Eileen Baildam, Louise Hanna, Olivia Lloyd, Susan Wadeson, Phil Riley, Ann McGovern, Clive Ryder, Janis Scott, Beverley Thomas, Taunton Southwood, Eslam Al‐Abadi, Sue Wyatt, Gillian Jackson, Tania Amin, Mark Wood, Vanessa VanRooyen, Deborah Burton, Joyce Davidson, Janet Gardner‐Medwin, Neil Martin, Sue Ferguson, Liz Waxman, Michael Browne, Mark Friswell, Helen Foster, Alison Swift, Sharmila Jandial, Vicky Stevenson, Debbie Wade, Ethan Sen, Eve Smith, Lisa Qiao, Stuart Watson, Claire Duong, Helen Venning, Rangaraj Satyapal, Elizabeth Stretton, Mary Jordan, Ellen Mosley, Anna Frost, Lindsay Crate, Kishore Warrier, Stefanie Stafford, Lucy Wedderburn, Clarissa Pilkington, Nathan Hasson, Sue Maillard, Elizabeth Halkon, Virginia Brown, Audrey Juggins, Sally Smith, Sian Lunt, Elli Enayat, Hemlata Varsani, Laura Kassoumeri, Laura Beard, Katie Arnold, Yvonne Glackin, Stephanie Simou, Beverley Almeida, Kiran Nistala, Shireena Yasin, Raquel Marques, Claire Deakin, Stefanie Dowle, Charis Papadopoulou, Cerise Johnson‐Moore, Emily Robinson, Kevin Murray, John Ioannou, Linda Suffield, Muthana Al‐Obaidi, Helen Lee, Sam Leach, Helen Smith, Anne‐Marie McMahon, Heather Chisem, Ruth Kingshott, Nick Wilkinson, Emma Inness, Eunice Kendall, David Mayers, Ruth Etherton, Danielle Miller, Kathryn Bailey, Jacqui Clinch, Natalie Fineman, Helen Pluess‐Hall, Lindsay Vallance, Louise Akeroyd, Alice Leahy, Amy Collier, Rebecca Cutts, Emma Macleod, Hans De Graaf, Brian Davidson, Sarah Hartfree, Danny Pratt

**Affiliations:** ^1^ University College London Great Ormond Street Institute of Child Health London UK; ^2^ Hospital de Santa Maria Centro Hospitalar Lisboa Norte Lisbon Academic Medical Centre and Instituto Português de Reumatologia Lisbon Portugal; ^3^ University College London Great Ormond Street Institute of Child Health Great Ormond Street Hospital for Children NIHR Biomedical Research Centre at Great Ormond Street Hospital for Children NHS Foundation Trust and Arthritis Research UK Centre for Adolescent Rheumatology at University College London University College London Hospital and Great Ormond Street Hospital London UK; ^4^ Hospital for Sick Children Toronto Ontario Canada; ^5^ Great Ormond Street Hospital for Children London UK

## Abstract

**Objective:**

In patients with severe or refractory juvenile dermatomyositis (DM), second‐line treatments may be required. Cyclophosphamide (CYC) is used to treat some connective tissue diseases, but evidence of its efficacy in juvenile DM is limited. This study was undertaken to describe clinical improvement in juvenile DM patients treated with CYC and model the efficacy of CYC treatment compared to no CYC treatment.

**Methods:**

Clinical data on skin, global, and muscle disease for patients recruited to the Juvenile DM Cohort and Biomarker Study were analyzed. Clinical improvement following CYC treatment was described using unadjusted analysis. Marginal structural models (MSMs) were used to model treatment efficacy and adjust for confounding by indication.

**Results:**

Compared to the start of CYC treatment, there were reductions at 6, 12, and 24 months in skin disease (*P* = 1.3 × 10^‐10^), global disease (*P* = 2.4 × 10^‐8^), and muscle disease (*P* = 8.0 × 10^‐10^) for 56 patients treated with CYC in unadjusted analysis. Limited evidence suggested a reduction in glucocorticoid dose (*P* = 0.047) in patients treated with CYC. MSM analysis showed reduced global disease and skin disease in patients who started an ~6‐month course of CYC treatment >12 months ago compared to patients never or not yet treated with CYC. In the treated patients, the modified skin Disease Activity Score for juvenile DM was 1.19 units lower (*P* = 0.0085) and the physician's global assessment was 0.66 units lower (*P* = 0.027). Minor adverse events were reported in 3 patients within 1 year of stopping CYC.

**Conclusion:**

Our findings indicate that CYC is efficacious with no short‐term side effects. Improvements in skin, global, and muscle disease were observed. Further studies are required to evaluate longer‐term side effects.

Juvenile dermatomyositis (DM) is a rare, severe autoimmune disease and the most prevalent idiopathic inflammatory myopathy in children, with an incidence of 2–4 cases per million per year 1. While some patients respond to standard first‐line treatment involving glucocorticoids and/or methotrexate, other patients may need second‐line treatments, for which limited data on treatment efficacy are available. Cyclophosphamide (CYC) is an alkylating agent that interferes with DNA replication and is toxic to rapidly dividing cells 2, 3. It is used to treat malignancy, severe systemic lupus erythematosus (SLE), and vasculitis 4, 5, 6, 7. Evidence to date of the efficacy of CYC for juvenile DM is based on case reports and a small descriptive study that showed sustained improvement in skin disease, particularly ulceration, and muscular and extramuscular features 8, 9, 10. There is international agreement that CYC is useful in severe or refractory disease 11; however, clinicians are often reluctant to give it because of possible side effects.

Evaluating the efficacy of second‐line treatments in rare diseases is challenging since conventional clinical trials may not be feasible, and it would take many years to recruit adequate patient numbers for a randomized controlled trial design. However, rare disease databases may provide a rich source of data for estimating treatment effects via an observational study. Drawing inferences about causality is challenging in observational studies, especially where there may be confounding by indication, whereby the clinical indication for selecting a certain medication also affects the outcome 12. For example, patients with more severe disease are more likely to be treated more aggressively but are also more likely to have poorer outcomes. Failure to adequately account for these confounding factors could lead to an incorrect conclusion that the more aggressive treatment leads to poorer outcomes. Indications for CYC usage in juvenile DM are severe or refractory disease, either at disease onset or later in the disease course. Severe disease is therefore a confounding factor associated with both CYC treatment and disease outcomes, and which changes over time. Marginal structural models (MSMs) are an analytical approach that can handle such time‐varying confounding factors 13. MSMs aim to balance the distribution of confounding covariates by generating ‘pseudo‐populations’ in which confounders and treatment assignment are unrelated. They achieve this by re‐weighting the data at each time point by inverse probability‐of‐treatment weighted estimators, which can take different values over time.

The aims of this study were to first, describe levels of clinical improvement in juvenile DM patients treated with CYC; and second, model the effect of CYC in patients who received CYC compared to patients who did not receive the drug, using an MSM approach.

## Patients and Methods

**Patients and clinical data.** Patients were recruited to the UK Juvenile DM Cohort and Biomarker Study (JDCBS), and written informed consent and age‐appropriate assent were obtained 14. (See Appendix A for members of the Juvenile DM Research Group.) The UK Northern & Yorkshire Medical Research & Ethics Committee approved this research (MREC/1/3/22). For all patients (n = 428 at the time of the study), data on demographic characteristics, all medications received, and clinical measures were collected at all recorded time points which corresponded to all clinical visits and were not necessarily at fixed intervals. Clinical measures included the modified Disease Activity Score (DAS) for assessing skin disease in juvenile DM (including Gottron's papules, heliotrope rash, vasculitis, and erythema) 15, 16, the physician's global assessment (PGA) of disease activity, and the Childhood Myositis Assessment Scale (CMAS) for assessment of muscle disease activity 17. Greater levels of disease severity are indicated by higher scores for the modified DAS for juvenile DM (range 0–5) and PGA (range 0–10) and lower scores on the CMAS (range 0–52). Myositis‐specific autoantibodies (MSAs) were measured 18, 19, 20, 21.

The following inclusion and exclusion criteria were necessary to exclude prevalent cases and retain incident cases for the MSM. The inclusion criterion was a full recorded history of disease, such that recorded visits spanned the whole of patients’ disease history and there were no major gaps in recorded history of early disease (prevalent cases). Exclusion criteria were time from diagnosis to first recorded visit exceeding 3 months and unknown date of starting CYC. Patients were not excluded from the MSM analysis if they were lost to follow‐up; however, such patients contributed fewer data points to the analytical model. These inclusion and exclusion criteria yielded 200 cases, comprising 56 patients treated with CYC and 144 patients not treated with CYC. Excluded patients did not differ in demographic features or diagnosis, and 15.4% received CYC (see Supplementary Table 1, available on the *Arthritis & Rheumatology* web site at http://onlinelibrary.wiley.com/doi/10.1002/art.40418/abstract).

**CYC treatment.** Internationally agreed upon indications for CYC are severe disease, including ulcerative skin disease and gastrointestinal disease causing perforation 11. Additional indications for CYC use at Great Ormond Street Hospital for Children (GOSH) include severe skin disease, severe muscle weakness, severe calcinosis, widespread vasculitis, and failure to respond to treatment. The current protocol for CYC treatment at GOSH, as the coordinating center with the most complete data, is 500 mg/m^2^ (maximum 500 mg) administered intravenously every 2 weeks for the first 3 doses and then 750 mg/m^2^ (maximum 1.2 gm) every 3–4 weeks according to the response for a total of 6–7 doses, with no further CYC infusions. Thus, a standardized noncontinuous treatment protocol was used, with the CYC treatment course completed within 6 months of initiation. The median duration of CYC treatment was 5.2 months (interquartile range [IQR] 4.6–6.5 months). There were no discontinuers.

**Statistical analysis.** *Statistical methods for descriptive analysis*. Statistical analysis was performed using R version 3.2.1 22. Data are presented as the number (percent) for categorical variables or the median (IQR) for numeric variables. Comparisons of demographic and clinical features of treatment groups were performed using the chi‐square test, Fisher's exact test, or Wilcoxon's signed rank test as appropriate. Improvements in clinical measures of disease activity over time at ~0, 6, 12, and 24 months after starting CYC treatment were analyzed using Friedman's test for nonparametric repeated‐measures analysis of variance. Since clinical visits did not occur at set time points, the nearest clinical measures to the 0, 6, 12, and 24 month time points were used, within ~2 months before or after the given time point, in order to maximize patient numbers and statistical power for this analysis. Exact times of clinical visits were used in the MSM analysis. Post hoc testing was performed using Wilcoxon's signed rank test to identify which time points were significantly different from each other in outcome measures following Friedman's test analysis. A Bonferroni correction was applied to adjust for multiple hypothesis testing.

*MSM analysis*. Full details of the MSM methodology are given in the Supplementary Methods, available on the *Arthritis & Rheumatology* web site at http://onlinelibrary.wiley.com/doi/10.1002/art.40418/abstract. Briefly, the MSM consists of first fitting a probability‐of‐treatment model (PoTM) to calculate inverse probability‐of‐treatment weights for use in a second analytical model. Longitudinal generalized estimating equation models were used for both the PoTM and final analytical model 23, 24. Although patients were only included if recorded visits spanned their whole disease history, PGA and CMAS values were not recorded at some visits. Therefore, prior to model fitting, multiple imputation was performed to impute missing values for the PGA and CMAS variables, generating 5 imputed data sets. All subsequent models and calculations for the PoTM and final analytical model of the MSM were performed separately for each imputed data set, with estimates from each analytical model for each imputed data set pooled as the final step.

The PoTM consisted of 3 separate models for: 1) probability at baseline of receiving CYC, 2) probability of starting CYC given not previously having been receiving CYC, and 3) probability of stopping CYC (Supplementary Tables 2–4, available on the *Arthritis & Rheumatology* web site at http://onlinelibrary.wiley.com/doi/10.1002/art.40418/abstract). None of the probabilities of treatment with CYC or propensity scores estimated by the PoTM were 0. Weights calculated using the estimates from the PoTM were used in the final analytical models, which modeled modified DAS for juvenile DM, PGA, and CMAS as outcomes. Predictor covariates in the final analytical model were time since diagnosis (to account for time effects) and a “split time” categorical variable, which was defined for the purpose of this study and was intended to capture the interaction between CYC and time, comprising “never/not yet received CYC,” “CYC within the last 6 months,” “CYC 6–12 months ago,” and “CYC >12 months ago.” The comparisons of interest were thus between patients who never received CYC (or had not yet initiated CYC) and patients who had initiated CYC either within the last 6 months (i.e., likely to still be receiving the CYC treatment course), or 6–12 months ago (i.e., recently completed the CYC treatment course), or >12 months ago (i.e., completed the CYC treatment course >6 months ago). The causal effect of interest in this study is therefore the continued effect of CYC treatment received over an ~6‐ month course.

## Results

**Demographic, clinical, and serologic features of patients receiving CYC and those not receiving CYC.** Both patients who received CYC and those who did not receive CYC were predominantly female, white, and diagnosed as having definite juvenile DM, and the 2 groups had an equivalent duration of follow‐up (Table 1). Patients who received CYC were younger at diagnosis than patients who did not receive CYC. When CYC was used, it was typically given within the first few months after diagnosis. Although skin disease activity at diagnosis was equivalent between the treatment groups, the patients treated with CYC had more global disease activity and muscle disease activity at diagnosis. This underscores the need for an MSM approach, since the patients treated with CYC had more severe disease. Proportions of the 4 more prevalent MSA subgroups did not differ between CYC and non‐CYC patients, indicating that severe disease, as the indication for CYC treatment, was present in all major MSA subgroups (Supplementary Table 5, available on the *Arthritis & Rheumatology* web site at http://onlinelibrary.wiley.com/doi/10.1002/art.40418/abstract). Notably, clinical scores and features associated with severe disease, including early interstitial lung disease, were present in both treatment groups (Table 1), indicating that the PoTM was fitted using patients representing a range of disease severities in both treatment groups, although the distribution was more skewed toward greater severity in the CYC group.

**Table 1:** Demographic and clinical features of patients treated with CYC compared to patients not treated with CYC^a^

	Treated with CYC (n = 56)	Not treated with CYC (n = 144)	*P* b
Sex			
Female	36 (64.3)	97 (67.4)	0.81^c^
Male	20 (35.7)	47 (32.6)	
Age at diagnosis, median (IQR) years	6.1 (4.0–9.7)	8.4 (5.2–12.0)	0.0051^d^
Ethnicity			
White	42 (75.0)	117 (81.3)	0.28^e^
Black	6 (10.7)	7 (4.9)	
South Asian	5 (8.9)	9 (6.3)	
Other	2 (3.6)	11 (7.6)	
Diagnosis^f^			
Definite juvenile DM	49 (87.5)	122 (84.7)	0.77^e^
Probable juvenile DM	4 (7.1)	11 (7.6)	
Juvenile DM overlapping with chronic polyarthritis	0 (0.0)	2 (1.4)	
Juvenile DM overlapping with mixed connective tissue disease	0 (0.0)	4 (2.8)	
Juvenile DM overlapping with scleroderma	3 (5.4)	5 (3.5)	
Duration of follow‐up, median (IQR) years	7.8 (4.6–10.7)	7.3 (4.2–11.2)	0.85^d^
Time from diagnosis to CYC start, median (IQR) days	33.5 (22–69.75)	–	–
Modified DAS for juvenile DM at baseline (first visit), median (IQR)	4 (4–5)	4 (3–5)	0.41^d^
PGA at baseline (first visit), median (IQR)	6.6 (4.1–8.0)	4.7 (2.7–7.0)	0.00074^d^
CMAS at baseline (first visit), median (IQR)	15.5 (5–29.75)	34 (17.5–46.5)	0.000091^d^
Patients with CMAS of 0 ever recorded during follow‐up	3 (5.4)	1 (0.7)	0.067^e^
Patients with calcinosis ever recorded during follow‐up	22 (39.3)	28 (19.4)	0.0064^c^
Patients with ulceration ever recorded during follow‐up	21 (37.5)	21 (14.6)	0.00073^c^
Patients with lipoatrophy ever recorded during follow‐up	13 (23.2)	18 (12.5)	0.096^c^
Patients with abnormal respiration ever recorded during follow‐up^g^	16 (28.6)	20 (13.9)	0.026^c^
Patients with edema ever recorded during follow‐up	36 (64.3)	68 (47.2)	0.044^c^

a Except where indicated otherwise, values are the number (%). CYC = cyclophosphamide; IQR = interquartile range; DAS = Disease Activity Score; PGA = physician's global assessment; CMAS = Childhood Myositis Assessment Scale.

b To adjust for multiple hypothesis testing, *P* values less than 0.0036 were considered statistically significant (Bonferroni correction applied to 14 independent tests).

c By chi‐square test.

d By Wilcoxon's signed rank test.

e By Fisher's exact test.

f Diagnosis of juvenile dermatomyositis (DM) was based on Bohan and Peter criteria 42, 43.

g Abnormal respiration, as a surrogate measure of early interstitial lung disease, included the following descriptions: shortness of breath, accessory muscle use, tachypnea, requires oxygen, and ventilated.

The large majority of patients in both treatment groups were treated with oral glucocorticoids and/or methotrexate (Table 2). Greater proportions of patients treated with CYC also received intravenous glucocorticoids and intravenous immunoglobulin, and there was a trend toward a higher proportion of these patients receiving hydroxychloroquine, infliximab, or adalimumab. Most patients within the CYC cohort who received these additional medications received them within the period beginning 6 months before CYC was started to 12 months after CYC was started. Therefore, these medications were included in the MSM to adjust for potential confounding effects.

**Table 2:** Additional medications received during follow‐up by patients treated with CYC and patients not treated with CYC^a^

	Treated with CYC (n = 56)	Not treated with CYC (n = 144)	*P* b
Oral glucocorticoids	54 (96.4)	124 (86.1)	0.066^c^
Intravenous glucocorticoids	36 (64.3)	58 (40.3)	0.0038^c^
Methotrexate	55 (98.2)	129 (89.6)	0.045^d^
Cyclosporine	0 (0)	0 (0)	–
Azathioprine	12 (21.4)	19 (13.2)	0.22^c^
Hydroxychloroquine	20 (35.7)	30 (20.8)	0.045^c^
Mycophenolate mofetil	6 (10.7)	8 (5.6)	0.22^d^
Intravenous immunoglobulin	18 (32.1)	18 (12.5)	0.0024^c^
Rituximab	1 (1.8)	1 (0.7)	0.48^d^
Etanercept	0 (0)	2 (1.4)	1^d^
Infliximab	16 (28.6)	16 (11.1)	0.0050^c^
Adalimumab	7 (12.5)	5 (3.5)	0.040^d^

a Values are the number (%). CYC = cyclophosphamide.

b To adjust for multiple hypothesis testing, *P* values less than 0.004 were considered statistically significant.

c By chi‐square test.

d By Fisher's exact test.

**Figure 1:**
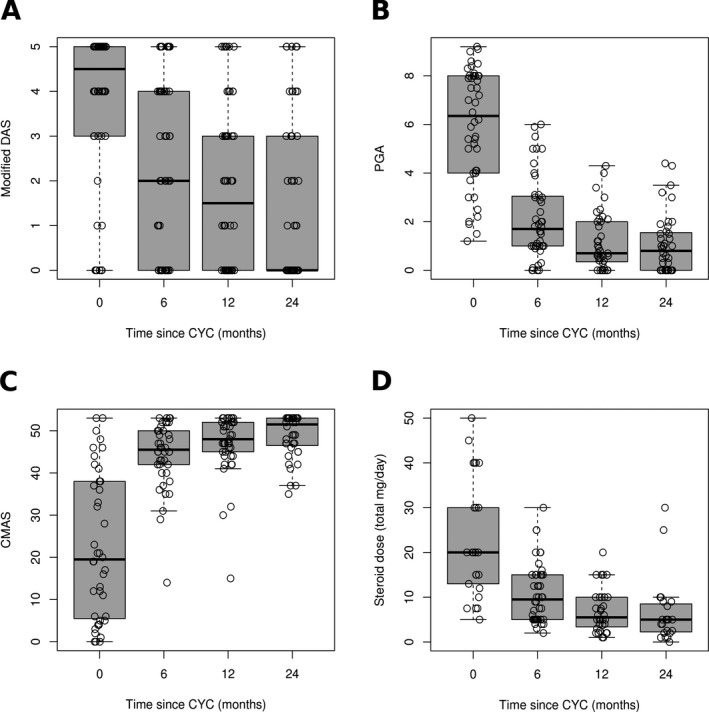
Improvement in **A**, modified Disease Activity Score (DAS) for juvenile dermatomyositis, **B**, physician's global assessment (PGA), **C**, Childhood Myositis Assessment Scale (CMAS), and **D**, glucocorticoid dose within 24 months of starting cyclophosphamide (CYC) treatment. Friedman's test for nonparametric repeated‐measures analysis of variance showed that overall, disease activity improved over the time points analyzed, with improvements in modified DAS for juvenile DM (χ^2^[3] = 49.0, *P* = 1.3 × 10^−10^), PGA (χ^2^[3] = 38.3, *P* = 2.4 × 10^‐8^), CMAS (χ^2^[3] = 45.3, *P* = 8.0 × 10^−10^), and glucocorticoid dose (χ^2^[3] = 7.9, *P* = 0.047). Percentages of missing data at 0, 6, 12, and 24 months, respectively, were as follows: 0%, 0%, 0%, and 0% for modified DAS for juvenile DM; 17.9%, 21.4%, 30.4%, and 28.6% for PGA; 28.6%, 25.0%, 23.2%, and 28.6% for CMAS; and 78.6%, 57.1%, 41.1%, and 44.6% for glucocorticoid dose. Circles represent individual patients; horizontal lines and boxes show the median (interquartile range). Bars above and below the boxes show the range.

**Improvement in skin disease, global disease, and muscle disease activities in patients treated with CYC.** Descriptive analysis at 0, 6, 12, and 24 months after the start of CYC treatment showed a significant reduction in skin disease activity (χ^2^[3] = 49.0, *P* = 1.3 × 10^−10^) (Figure 1A). The median modified DAS for juvenile DM decreased from 4.5 (IQR 3–5) at the start of CYC treatment to 2 (IQR 0–4) at 6 months (*P* = 3.5 × 10^−5^), 1.5 (IQR 0–3) at 12 months (*P* = 2.6 × 10^−6^), and 0 (IQR 0–3) at 24 months (*P* = 6.2 × 10^−7^). The modified DAS for juvenile DM at 24 months was also significantly lower than at 6 months (*P* = 0.003). There was also a significant reduction in global disease activity (χ^2^[3] = 38.3, *P* = 2.4 × 10^−8^) (Figure 1B). The median PGA decreased from 6.4 (IQR 4.0–8.0) at the start of CYC treatment to 1.7 (IQR 1.0–3.0) at 6 months (*P* = 5.8 × 10^−7^), 0.7 (IQR 0.4–2.0) at 12 months (*P* = 2.8 × 10^−6^), and 0.8 (IQR 0.0–1.5) at 24 months (*P* = 3.8 × 10^−7^). PGA at 24 months was also significantly lower than at 6 months (*P* = 0.008). Muscle disease activity also significantly improved (χ^2^[3] = 45.3, *P* = 8.0 × 10^−10^) (Figure 1C). The median CMAS increased from 19.5 (IQR 5.75–38) at the start of CYC treatment to 45.5 (IQR 42–50) at 6 months (*P* = 2.0 × 10^‐6^), 48 (IQR 44–52) at 12 months (*P* = 4.0 × 10^−6^), and 51.5 (IQR 46.75–52) at 24 months (*P* = 8.3 × 10^−6^). CMAS at 12 months and 24 months were also significantly higher than at 6 months (*P* = 0.0002 and *P* = 0.0002, respectively).

There also appeared to be a significant reduction in glucocorticoid dose in patients treated with CYC (χ^2^[3] = 7.9, *P* = 0.047) (Figure 1D). The median (IQR) glucocorticoid dose decreased from 20 mg/day (IQR 13–30) at baseline to 9.5 mg/day (IQR 5–15) at 6 months (*P* = 0.0004), 5.5 mg/day (IQR 3.6–10) at 12 months (*P* = 0.0009), and 5 mg/day (IQR 2.3–8.5) at 24 months (*P* = 0.006). The median glucocorticoid doses at 12 months and 24 months were also significantly lower than at 6 months (*P* = 0.004 and *P* = 0.007, respectively). However, 41.1–78.6% of precise glucocorticoid dose data were missing at the visits corresponding to these time points, compared to no missing data for modified DAS for juvenile DM, 17.9–30.4% missing for PGA, and 23.2–28.6% missing for CMAS. It is possible that the pattern of missing glucocorticoid dose data could reflect an underlying systematic bias, which may limit the representativeness of the finding of a reduction in glucocorticoid dose. It therefore may be more reasonable to interpret these data as suggesting a downward trend in glucocorticoid dose.

**Figure 2:**
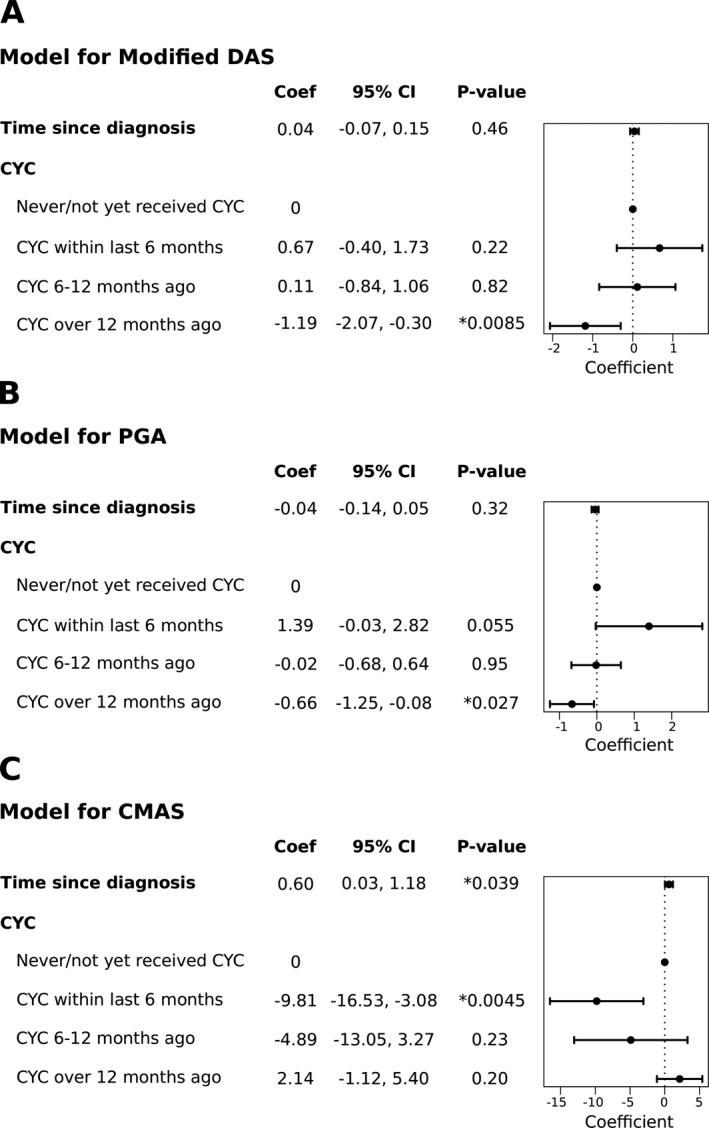
Longitudinal marginal structural model (MSM) analysis of cyclophosphamide (CYC) efficacy for improvement in juvenile dermatomyositis (DM). Forest plots depict estimated regression coefficients (Coef) from final analytical models where modified Disease Activity Score (DAS) for juvenile DM (**A**), physician's global assessment (PGA) (**B**), and Childhood Myositis Assessment Scale (CMAS) (**C**) were modeled as outcomes using data from 56 patients treated with CYC and 144 patients not treated with CYC. These final analytical models were weighted using inverse propensity score values generated by an MSM, in order to balance confounding differences between patients who were and those who were not treated with CYC. **Asterisks** indicate significant *P* values. 95% CI = 95% confidence interval.

**MSM analysis of CYC efficacy.** An MSM approach was used to model the efficacy of CYC treatment on skin disease, global disease, and muscle disease activities over time (Figure 2). In the analytical model for skin disease, there was no effect for patients who were treated with CYC either in the last 6 months or 6–12 months ago, relative to patients who were never or not yet treated with CYC (Figure 2A). However, patients who received CYC >12 months ago had reduced skin disease activity over time. The modified DAS for juvenile DM was on average 1.19 units lower in these patients compared to patients who were never or not yet treated with CYC (*P* = 0.0085).

The analytical model for global disease activity showed a trend toward increased disease activity in patients who received CYC within the last 6 months, compared to patients who were never or not yet treated with CYC (Figure 2B). CYC did not have an effect on global disease activity in patients who received the drug 6–12 months ago. Similar to the analytical model for the modified DAS for juvenile DM, CYC was efficacious in patients who received CYC >12 months ago compared to patients who had never received or were not yet treated with CYC. PGA was on average 0.66 units lower in these patients (*P* = 0.027).

In the analytical model for muscle disease activity, CYC did not result in a significant improvement (Figure 2C). CMAS was 9.81 units lower, indicating higher disease activity, in patients who received CYC within the last 6 months compared to patients who never or had not yet received CYC. CYC had no effect for patients who received CYC 6–12 months ago, but there was a trend toward improvement in patients treated with CYC >12 months ago.

Plots of weighted and unweighted mean modified DAS for juvenile DM, PGA, and CMAS for the first 2 years after diagnosis and for both the patients who had never or not yet received CYC and the patients at the start of CYC treatment showed that at some, but not all, time points weighting improved the balance between these 2 treatment groups, as indicated by time points where the confidence intervals of the weighted means for the treatment groups overlapped (Supplementary Figure 1, available on the *Arthritis & Rheumatology* web site at http://onlinelibrary.wiley.com/doi/10.1002/art.40418/abstract). This residual confounding could account for the increased disease activity observed within the first 12 months of CYC treatment in all 3 analytical models.

**Minimal side effects of CYC.** Minor adverse events were reported in 3 patients within 1 year of stopping CYC; these were 3 respiratory infections and 1 episode of mouth ulcers. There were a further 4 reported adverse events, but these occurred >1 year after stopping CYC. There were no reports of infertility or malignancy, although the follow‐up data available at present are insufficient to fully evaluate these issues.

## Discussion

This study used both a descriptive analysis and an MSM approach to investigate the efficacy of CYC retrospectively using a large national cohort of juvenile DM patients. In the descriptive analysis, improvements in global disease, skin disease, and muscle disease activity following CYC treatment were found. In the MSM analysis, the greatest benefit was observed in skin disease and global disease activity at 12 months after the start of CYC treatment, relative to patients who never or had not yet received CYC. Delay to diagnosis is linked to poor prognosis in juvenile DM 25, and aggressive treatment at an early stage of disease leads to better outcomes 11. We found that CYC has sustained beneficial effects, which could possibly be related to an effect of CYC in resetting the immunologic milieu, such that disease is downgraded. Long‐term beneficial effects of CYC treatment for lupus nephritis have also been reported 26. Improvement in juvenile DM skin disease was noted previously 10.

At present, longer‐term side effects of CYC on fertility and malignancy remain unknown and need to be addressed by further studies. The cumulative doses of CYC used in this study are below levels likely to cause either of these complications. When similar or higher doses of CYC have been administered to women with SLE, younger age (especially younger than 25 years) has had a protective effect against infertility issues 27, 28, 29. Infertility is rare in males who have received doses lower than 7.5 gm/m^2^ 30, 31, 32. Malignancy has been reported in one 17‐year‐old female treated with a cumulative dose of >20 gm of CYC 33, and otherwise a limited number of malignancies have developed in adult SLE patients, who typically received higher doses of CYC 34, 35, 36. Initially at GOSH, the standard NIH protocol for CYC treatment of SLE of 12 doses was applied to juvenile DM 37, 38, 39; however, since noticeable improvement was observed after 6 doses, the protocol was changed over the years to the current protocol of giving a maximum of 6–7 doses. It remains unknown whether further reducing the dose to, for example, the first 4 doses could retain a high degree of efficacy while further improving the safety profile.

In rare diseases, establishing clinical trials to evaluate drug efficacy is particularly challenging. This study highlights the value of long‐term observational studies in rare diseases and the use of analytical methods to maximize the knowledge that can be derived from such studies. Data from a large number of patients recruited to the UK‐wide JDCBS enabled a study of the largest‐ever analyzed cohort of juvenile DM patients treated with CYC. The MSM approach enabled these patients to be directly compared with patients who had never or not yet received CYC by accounting for confounding by indication.

MSMs yield unbiased causal estimates of treatment effects provided certain assumptions are met. First, there should be no unmeasured confounders, so that the data set captures all of the factors predictive of both outcomes and treatment with CYC. Second, there must be some unexplained variation in who gets CYC at any given time point; this is known as the experimental treatment assignment assumption. Thus, MSMs are not suitable for studies where treatment is strictly protocolized. Even after applying inverse probability‐of‐treatment weights, the confidence intervals for the mean modified DAS for juvenile DM, PGA, and CMAS did not overlap at certain time points, for both the patients who had never or not yet received CYC and the patients at the start of CYC treatment. This suggests that confounding of these treatment groups was not perfectly controlled for by the MSM, particularly within the first 12 months, suggesting unexplained variation in CYC initiation and the possibility of unmeasured confounders. Consequently, it is possible that the MSM analysis underestimated CYC efficacy. Some indications for CYC use, such as failure to respond to standard treatment and vasculitis, may have been inadequately captured by the variables used in the PoTM.

The analytical approach of this observational study also has limitations. The inverse probability‐of‐treatment weights used in MSMs often have a very skewed distribution. This can lead to the undesirable scenario where the results are driven by a single observation, or by a single patient. For this reason, truncation of the weights is recommended 40, 41, and 20 is often chosen as the truncation point. Following this practice, we truncated our weights at 20; however, we recognize that the truncation point is arbitrary.

The findings of improved clinical benefit within the first 12 months of CYC treatment in the descriptive analysis appear to conflict with the results of MSM analysis, which show either no improvement or an increase in disease activity within the first 12 months of CYC treatment and improvement in skin and global disease after the first 12 months. It is possible that patients who did not receive CYC also showed clinical benefit over that time period, thus lessening the relative benefit in the patients treated with CYC during the first 12 months, with relative benefit being most apparent after those first 12 months. It is also possible the MSM did not adequately control for confounding during this time period, such that treatment efficacy was underestimated.

In summary, we have shown that CYC is efficacious with no reported side effects using a large national cohort of juvenile DM patients. In contrast to biologic therapies that are also used as second‐line treatments in juvenile DM, CYC is an inexpensive drug requiring a short treatment course of 6–7 months. As long as infections are monitored, the sustained clinical benefit demonstrated by this study suggests that more frequent use of CYC may be warranted.

## Author contributions

All authors were involved in drafting the article or revising it critically for important intellectual content, and all authors approved the final version to be published. Dr. Deakin had full access to all of the data in the study and takes responsibility for the integrity of the data and the accuracy of the data analysis.

### Study conception and design

Deakin, Campanilho‐Marques, Moraitis, Wedderburn, Pilkington.

### Acquisition of data

Deakin, Campanilho‐Marques, Simou, Wedderburn, Pilkington.

### Analysis and interpretation of data

Deakin, Campanilho‐Marques, Pullenayegum, Pilkington.

## Appendix A Juvenile Dermatomyositis Research Group Members

The following Juvenile Dermatomyositis Research Group members contributed to the study: Dr. Kate Armon, Mr. Joe Ellis‐Gage, Ms Holly Roper, Ms Vanja Briggs, Ms Joanna Watts (Norfolk and Norwich University Hospitals); Dr. Liza McCann, Mr. Ian Roberts, Dr. Eileen Baildam, Ms Louise Hanna, Ms Olivia Lloyd, Ms Susan Wadeson (The Royal Liverpool Children's Hospital, Alder Hey, Liverpool); Dr. Phil Riley, Ms Ann McGovern (Royal Manchester Children's Hospital, Manchester); Dr. Clive Ryder, Mrs. Janis Scott, Mrs. Beverley Thomas, Professor Taunton Southwood, Dr. Eslam Al‐Abadi (Birmingham Children's Hospital, Birmingham); Dr. Sue Wyatt, Mrs. Gillian Jackson, Dr. Tania Amin, Dr. Mark Wood, Dr. Vanessa VanRooyen, Ms Deborah Burton (Leeds General Infirmary, Leeds); Dr. Joyce Davidson, Dr. Janet Gardner‐Medwin, Dr. Neil Martin, Ms Sue Ferguson, Ms Liz Waxman, Mr. Michael Browne (The Royal Hospital for Sick Children, Yorkhill, Glasgow); Dr. Mark Friswell, Professor Helen Foster, Mrs. Alison Swift, Dr. Sharmila Jandial, Ms Vicky Stevenson, Ms Debbie Wade, Dr. Ethan Sen, Dr. Eve Smith, Ms Lisa Qiao, Mr. Stuart Watson, Ms Claire Duong (Great North Children's Hospital, Newcastle); Dr. Helen Venning, Dr. Rangaraj Satyapal, Mrs. Elizabeth Stretton, Ms Mary Jordan, Dr. Ellen Mosley, Ms Anna Frost, Ms Lindsay Crate, Dr. Kishore Warrier, Ms Stefanie Stafford (Queens Medical Centre, Nottingham); Professor Lucy Wedderburn, Dr. Clarissa Pilkington, Dr. Nathan Hasson, Mrs. Sue Maillard, Ms Elizabeth Halkon, Ms Virginia Brown, Ms Audrey Juggins, Dr. Sally Smith, Mrs. Sian Lunt, Ms Elli Enayat, Mrs. Hemlata Varsani, Miss Laura Kassoumeri, Miss Laura Beard, Miss Katie Arnold, Mrs. Yvonne Glackin, Ms Stephanie Simou, Dr. Beverley Almeida, Dr. Kiran Nistala, Dr. Shireena Yasin, Dr. Raquel Marques, Dr. Claire Deakin, Ms Stefanie Dowle, Dr. Charis Papadopoulou, Mrs. Cerise Johnson‐Moore, Ms Emily Robinson (Great Ormond Street Hospital, London); Dr. Kevin Murray (Princess Margaret Hospital, Perth, Western Australia); Dr. John Ioannou, Ms Linda Suffield (University College London Hospital, London); Dr. Muthana Al‐Obaidi, Ms Helen Lee, Ms Sam Leach, Ms Helen Smith, Dr. Anne‐Marie McMahon, Ms Heather Chisem, Ms Ruth Kingshott (Sheffield's Children's Hospital, Sheffield); Dr. Nick Wilkinson, Ms Emma Inness, Ms Eunice Kendall, Mr. David Mayers, Ms Ruth Etherton, Ms Danielle Miller, Dr. Kathryn Bailey (Oxford University Hospitals, Oxford); Dr. Jacqui Clinch, Ms Natalie Fineman, Ms Helen Pluess‐Hall (Bristol Royal Hospital for Children, Bristol); Ms Lindsay Vallance (Royal Aberdeen Children's Hospital); Ms Louise Akeroyd (Bradford Teaching Hospitals); and Dr Alice Leahy, Ms Amy Collier, Ms Rebecca Cutts, Ms Emma Macleod, Dr. Hans De Graaf, Dr. Brian Davidson, Ms Sarah Hartfree, Mr. Danny Pratt (University Hospital Southampton).
