# Supplementary material for: Efficacy and Safety of Cyclophosphamide Treatment in Severe Juvenile Dermatomyositis Shown by Marginal Structural Modeling
Source: Arthritis Rheumatol. 2018 Mar 25;70(5):785–93. doi: 10.1002/art.40418 (PMC5947636; doi:10.1002/art.40418)
Supplement: Supplementary file 1 [file ART-70-785-s001.docx]

**SUPPLEMENTARY METHODS**

*Full details of MSM methodology*

Due to the presence of missing clinical data for PGA and CMAS at 15%-20% of time-points, multiple imputation was performed using the “Amelia” package in R version 3.2.1 [1]. Five imputed datasets were generated and the variables used for imputation were: time since diagnosis, modified DAS, PGA, CMAS, CYC received ever, on CYC at the specific time-point, baseline modified DAS, baseline PGA, baseline CMAS, age at diagnosis, ulceration, calcinosis and oedema. The modified DAS was calculated based on whether erythema, Gottron’s papules, Heliotrope rash and vasculitis were present [2]. Where any of these variables were missing they were treated as absent in order to avoid the need to impute this variable. However, this calculated modified DAS may consequently underestimate the true modified DAS.

The fitting of the probability-of-treatment model (PoTM) and the final analytical model were performed separately for each of the 5 imputed datasets. Estimates from the final analytical models for each imputed dataset were combined as the final step of the method. The PoTM consisted of 3 separate models covering the time-points indicated: a baseline model (time-point at or closest to diagnosis), a starting CYC model (time-points after baseline up to the first time-point on CYC), and a stopping CYC model (second time-point on CYC up to the first time-point off CYC). The baseline model purports to achieve balance across confounding covariates between treatment groups at baseline (diagnosis). The starting CYC model purports to achieve balance between new initiators of CYC and non-initiators of CYC. The stopping CYC model purports to achieve balance between discontinuers of CYC and continuers of CYC. Fitting each of these 3 separate models involved a separate “for” loop to loop across each of the 5 imputed datasets. Logistic regression using the “glm” function in R was used to fit the baseline model, which modelled the probability of receiving CYC ever. Covariates for the baseline model were: modified DAS, PGA and CMAS. Estimates and standard errors for these covariates are reported in Supplementary Table 2.

The starting CYC model was a generalized estimating equations (GEE) model, which is a longitudinal method for modelling recurrent events [3]. All GEE models in this study used either the “geeglm()” function or “geese()” function in the R package “geepack” and an independent correlation structure [4]. The starting CYC model modelled the probability of being on CYC at each time-point after baseline up to the first time-point on CYC for the CYC patients, and all time-points after baseline for the non-CYC patients. Covariates for this model were: time since diagnosis, modified DAS, PGA, CMAS, age, ulceration, calcinosis, oedema, infliximab, intravenous immunoglobulin, azathioprine, and intravenous steroids. The time since diagnosis variable was truncated at 12 months as this helped to minimize extreme weights. For the first on-CYC time-point, covariate values were lagged by 1 such that the values from the previous visit were used as the previous visit. This was felt to more closely reflect the clinical circumstances indicating CYC use. Additional covariates were used in the starting CYC model to optimize model fit and thus improve balance between treatment groups. Estimates and standard errors for these covariates are reported in Supplementary Table 3. The stopping CYC model was also a GEE model and modelled the probability of being off CYC at each time-point from the second on-CYC time-point until the first off-CYC time-point, and used data for just the CYC patients. Covariates for this model were: time since diagnosis, modified DAS, PGA and CMAS. For the first off-CYC time-point, covariate values were lagged by 1 such that the values from the previous visit were used. This was similarly done to more closely reflect clinical circumstances indicating stopping CYC. Estimates and standard errors for these covariates are reported in Supplementary Table 4. All covariates used in each of the baseline, starting CYC and stopping CYC models were time-varying. Weight stabilization was not used because there is some evidence that the usual stabilized weights can lead to bias [5]. Although stabilizers containing time invariant variables could have been used, since these would have needed to be in the final analytical model as well, the flexibility of not needing to include them in the final analytical model was preferred.

The estimated coefficients generated by each of the baseline, starting CYC and stopping CYC models of the PoTM were used to calculate inverse propensity scores at each time-point for each of the 5 imputed datasets as follows. The model estimates were used to calculate log odds and hence predicted probability of treatment. Probability of *not* receiving treatment was calculated as 1 minus the predicted probability of treatment. The propensity score for the CYC patients was thus the predicted probability of treatment, and the propensity score for the patients who did not receive CYC was thus the probability of not receiving treatment. None of the probabilities of treatment with CYC or propensity scores estimated by the PoTM were zero, thus satisfying one of the key assumptions of the MSM method (positivity violation) [6]. The inverse propensity score was then the reciprocal of these propensity scores. For patients on CYC, at any time-points after the first time-point off CYC the previous value for the inverse propensity score was carried forward. Finally, to minimize extreme values, all calculated inverse propensity scores were normalized to 1 by dividing by the mean score value, as a means of taking sample size into account, and then these normalized inverse propensity scores were truncated at 20. This normalization made no difference to the results of the final analytical model, since it is the relative weights that matter, but it facilitates interpretation of what constitutes a large weight.

To check whether these normalized inverse propensity scores achieved balance between the treatment groups, the weighted means of the modified DAS, PGA and CMAS were compared. For each imputed dataset, time-points at intervals of 0.1 years were aggregated up to 2 years since diagnosis for patients in the CYC group at the time-point closest to when CYC was started (where this was no longer than 6 months before or after CYC start) and for patients who never or not yet received CYC. At each of these intervals within each imputed dataset, weighted and unweighted means and standard errors were calculated using the “wtd.mean()” and “wtd.var()” functions in the R package “Hmisc”. Estimates for weighted and unweighted means and standard errors for each time-point interval were pooled using the “mi.meld()” function in “Amelia”. The estimated means for each time-point and a 95% confidence interval for each estimate were plotted using the “plotCI()” function from the R package “gplots” [7].

The final analytical models were fitted separately for each imputed dataset. These were also GEE models with independent correlation structures, and separate models were fitted for the 3 outcomes of interest: modified DAS, PGA and CMAS. The same predictor variables were used across all of the final analytical models: time since diagnosis and a split-time variable consisting of the categories “never/not yet received CYC”, “CYC within the last 6 months”, “CYC 6-12 months ago” and “CYC over 12 months ago”. The normalized inverse propensity scores that were calculated using the PoTM estimates were used as weights in the final analytical models.

Finally, the estimates from the final analytical models fitted across the 5 imputed datasets were pooled using the “mi.meld()” function in “Amelia”. Forest plots of the pooled estimated effects of each covariate in the analytical models and 95% confidence intervals for those estimates were produced using the “plotCI” function in the R package “gplots”. The CYC treatment group represents patients starting CYC and the non-CYC group represents patients who had never or not yet received CYC. In the CYC group, data points without confidence intervals represent time-points where just 1 patient started CYC. Patients treated with CYC in this study tended to initiate CYC within the first 6 months of diagnosis (Table 1).

**SUPPLEMENTARY RESULTS**

**Supplementary Table 1. Features of patients included and excluded from study^a^**

|  | **Patients included in study (n=200)** | **Patients excluded in study (n=228)** | **P-value** |
| --- | --- | --- | --- |
| **Number on CYC** | 56 (28.0%) | 35 (15.4%)) | 0.0021^b^ |
| **Sex** |  |  |  |
| Female | 133 (66.5%) | 167 (73.2%) | 0.16^b^ |
| Male | 67 (33.5%) | 61 (26.8%) |  |
| **Age at diagnosis (years)** | 7.6 [4.8-11.1] | 7.0 [4.4-10.5] | 0.21^c^ |
| **Diagnosis^e^** |  |  |  |
| Definite JDM | 171 (85.5%) | 183 (80.3%) | 0.17^d^ |
| Probable JDM | 15 (7.5%) | 14 (6.1%) |  |
| JDM overlap with scleroderma | 8 (4.0%) | 17 (7.4%) |  |
| JDM overlap with mixed connective tissue disease | 4 (2.0%) | 4 (1.8%) |  |
| JDM overlap with chronic polyarthritis | 2 (1.0%) | 4 (1.8%) |  |
| JDM overlap with systemic lupus erythematosus | 0 (0.0%) | 5 (2.2%) |  |

^a^Data are presented as number (percentage) for categorical variables or median (interquartile range) for numeric variables

^b^Analysis using Chi-square test

^c^Analysis using Wilcoxon signed rank test

^d^Analysis using Fisher’s exact test

^e^Diagnosis with juvenile dermatomyositis (JDM) according to Bohan and Peter criteria [8,9].

**Supplementary Table 2. Regression coefficients for models comprising the baseline model of the probability-of-treatment model fitted to each imputed dataset**

| **Imputed dataset** | **Covariate** | **Estimate** | **Standard error** | **Z value** | **P-value** |
| --- | --- | --- | --- | --- | --- |
| **1** | **Intercept** | -0.73 | 1.02 | -0.72 | 0.47 |
|  | **Modified DAS** | -0.07 | 0.15 | -0.47 | 0.64 |
|  | **PGA** | 0.06 | 0.10 | 0.56 | 0.57 |
|  | **CMAS** | -0.03 | 0.02 | -1.98 | 0.05 |
| **2** | **Intercept** | -0.62 | 0.99 | -0.63 | 0.53 |
|  | **Modified DAS** | -0.05 | 0.15 | -0.37 | 0.71 |
|  | **PGA** | 0.03 | 0.10 | 0.30 | 0.76 |
|  | **CMAS** | -0.03 | 0.02 | -2.13 | 0.03 |
| **3** | **Intercept** | -0.089 | 0.99 | -0.91 | 0.37 |
|  | **Modified DAS** | -0.05 | 0.15 | -0.32 | 0.75 |
|  | **PGA** | 0.04 | 0.10 | 0.43 | 0.67 |
|  | **CMAS** | -0.03 | 0.02 | -1.67 | 0.09 |
| **4** | **Intercept** | -0.52 | 0.96 | -0.54 | 0.59 |
|  | **Modified DAS** | -0.05 | 0.15 | -0.31 | 0.76 |
|  | **PGA** | 0.01 | 0.10 | 0.14 | 0.89 |
|  | **CMAS** | -0.04 | 0.02 | -2.26 | 0.02 |
| **5** | **Intercept** | -0.78 | 0.99 | -0.79 | 0.43 |
|  | **Modified DAS** | -0.05 | 0.15 | -0.33 | 0.74 |
|  | **PGA** | 0.05 | 0.10 | 0.48 | 0.63 |
|  | **CMAS** | -0.03 | 0.02 | -1.98 | 0.05 |

CMAS, Childhood Myositis Assessment Scale; DAS, Disease Activity Score; PGA, physician’s global assessment of disease activity

**Supplementary Table 3. Regression coefficients for models comprising the starting CYC model of the probability-of-treatment model fitted to each imputed dataset**

| **Imputed dataset** | **Covariate** | **Estimate** | **Standard error** | **Z value** | **P-value** |
| --- | --- | --- | --- | --- | --- |
| **1** | **Intercept** | -2.66 | 1.42 | 3.51 | 0.06 |
|  | **Time since diagnosis** | -2.80 | 0.85 | 10.81 | 0.00 |
|  | **Modified DAS** | 0.27 | 0.19 | 2.19 | 0.14 |
|  | **PGA** | 0.11 | 0.13 | 0.69 | 0.41 |
|  | **CMAS** | -0.02 | 0.02 | 1.05 | 0.31 |
|  | **Age** | 0.06 | 0.05 | 1.66 | 0.20 |
|  | **Ulceration** | 0.78 | 0.58 | 1.80 | 0.18 |
|  | **Calcinosis** | 0.30 | 0.66 | 0.21 | 0.65 |
|  | **Oedema** | 0.42 | 0.56 | 0.56 | 0.45 |
|  | **IVIG** | 0.14 | 0.74 | 0.03 | 0.85 |
|  | **Azathioprine** | 0.30 | 0.88 | 0.11 | 0.74 |
|  | **Infliximab** | 0.45 | 1.03 | 0.19 | 0.66 |
|  | **IV steroids** | 0.81 | 0.53 | 2.33 | 0.13 |
| **2** | **Intercept** | -2.58 | 1.38 | 3.50 | 0.06 |
|  | **Time since diagnosis** | -2.80 | 0.87 | 10.47 | 0.00 |
|  | **Modified DAS** | 0.27 | 0.19 | 2.14 | 0.14 |
|  | **PGA** | 0.12 | 0.13 | 0.85 | 0.36 |
|  | **CMAS** | -0.02 | 0.02 | 1.38 | 0.24 |
|  | **Age** | 0.06 | 0.05 | 1.86 | 0.17 |
|  | **Ulceration** | 0.48 | 0.51 | 0.89 | 0.35 |
|  | **Calcinosis** | 0.02 | 0.59 | 0.00 | 0.97 |
|  | **Oedema** | 0.49 | 0.53 | 0.84 | 0.36 |
|  | **IVIG** | 0.10 | 0.75 | 0.02 | 0.90 |
|  | **Azathioprine** | 0.28 | 0.97 | 0.09 | 0.77 |
|  | **Infliximab** | 0.53 | 1.05 | 0.26 | 0.61 |
|  | **IV steroids** | 0.80 | 0.53 | 2.28 | 0.13 |
| **3** | **Intercept** | -2.65 | 1.50 | 3.14 | 0.08 |
|  | **Time since diagnosis** | -2.84 | 0.83 | 11.79 | 0.00 |
|  | **Modified DAS** | 0.29 | 0.19 | 2.35 | 0.13 |
|  | **PGA** | 0.08 | 0.15 | 0.27 | 0.60 |
|  | **CMAS** | -0.02 | 0.02 | 0.86 | 0.35 |
|  | **Age** | 0.06 | 0.05 | 1.33 | 0.25 |
|  | **Ulceration** | 0.52 | 0.53 | 0.94 | 0.33 |
|  | **Calcinosis** | 0.65 | 0.53 | 1.51 | 0.22 |
|  | **Oedema** | 0.71 | 0.54 | 1.73 | 0.19 |
|  | **IVIG** | 0.14 | 0.74 | 0.03 | 0.85 |
|  | **Azathioprine** | 0.33 | 0.91 | 0.13 | 0.72 |
|  | **Infliximab** | 0.34 | 1.03 | 0.11 | 0.74 |
|  | **IV steroids** | 0.86 | 0.54 | 2.56 | 0.11 |
| **4** | **Intercept** | -3.00 | 1.41 | 4.50 | 0.03 |
|  | **Time since diagnosis** | -2.86 | 0.87 | 10.74 | 0.00 |
|  | **Modified DAS** | 0.29 | 0.18 | 2.48 | 0.12 |
|  | **PGA** | 0.14 | 0.13 | 1.19 | 0.28 |
|  | **CMAS** | -0.01 | 0.02 | 0.44 | 0.51 |
|  | **Age** | 0.06 | 0.05 | 1.59 | 0.21 |
|  | **Ulceration** | 0.30 | 0.53 | 0.31 | 0.58 |
|  | **Calcinosis** | -0.03 | 0.57 | 0.00 | 0.96 |
|  | **Oedema** | 0.44 | 0.55 | 0.64 | 0.43 |
|  | **IVIG** | 0.14 | 0.72 | 0.04 | 0.85 |
|  | **Azathioprine** | 0.24 | 0.99 | 0.06 | 0.81 |
|  | **Infliximab** | 0.53 | 1.05 | 0.25 | 0.61 |
|  | **IV steroids** | 0.86 | 0.52 | 2.72 | 0.10 |
| **5** | **Intercept** | -2.65 | 1.48 | 3.19 | 0.07 |
|  | **Time since diagnosis** | -2.83 | 0.86 | 10.82 | 0.00 |
|  | **Modified DAS** | 0.29 | 0.19 | 2.40 | 0.12 |
|  | **PGA** | 0.09 | 0.14 | 0.43 | 0.51 |
|  | **CMAS** | -0.02 | 0.02 | 1.06 | 0.30 |
|  | **Age** | 0.07 | 0.05 | 2.10 | 0.15 |
|  | **Ulceration** | 0.76 | 0.57 | 1.78 | 0.18 |
|  | **Calcinosis** | -0.08 | 0.62 | 0.02 | 0.90 |
|  | **Oedema** | 0.41 | 0.56 | 0.56 | 0.46 |
|  | **IVIG** | 0.20 | 0.74 | 0.07 | 0.79 |
|  | **Azathioprine** | 0.26 | 0.92 | 0.08 | 0.78 |
|  | **Infliximab** | 0.47 | 1.02 | 0.21 | 0.65 |
|  | **IV steroids** | 0.83 | 0.54 | 2.33 | 0.13 |

CMAS, Childhood Myositis Assessment Scale; DAS, Disease Activity Score; IV, intravenous; IVIG, intravenous immunoglobulin; PGA, physician’s global assessment of disease activity

**Supplementary Table 4. Regression coefficients for models comprising the starting CYC model of the probability-of-treatment model fitted to each imputed dataset**

| **Imputed dataset** | **Covariate** | **Estimate** | **Standard error** | **Z value** | **P-value** |
| --- | --- | --- | --- | --- | --- |
| **1** | **Intercept** | 5.35 | 1.92 | 7.76 | 0.01 |
|  | **Time since diagnosis** | -0.37 | 0.38 | 0.94 | 0.33 |
|  | **Modified DAS** | 0.06 | 0.13 | 0.18 | 0.67 |
|  | **PGA** | -0.09 | 0.21 | 0.21 | 0.65 |
|  | **CMAS** | -0.12 | 0.04 | 8.01 | 0.00 |
| **2** | **Intercept** | 3.98 | 1.72 | 5.35 | 0.02 |
|  | **Time since diagnosis** | -0.39 | 0.51 | 0.59 | 0.44 |
|  | **Modified DAS** | 0.02 | 0.12 | 0.03 | 0.86 |
|  | **PGA** | -0.04 | 0.20 | 0.03 | 0.86 |
|  | **CMAS** | -0.09 | 0.04 | 5.16 | 0.02 |
| **3** | **Intercept** | 4.27 | 1.86 | 5.30 | 0.02 |
|  | **Time since diagnosis** | -0.43 | 0.52 | 0.71 | 0.40 |
|  | **Modified DAS** | 0.08 | 0.12 | 0.41 | 0.52 |
|  | **PGA** | -0.06 | 0.18 | 0.10 | 0.75 |
|  | **CMAS** | -0.09 | 0.04 | 5.07 | 0.02 |
| **4** | **Intercept** | 5.14 | 2.00 | 6.60 | 0.01 |
|  | **Time since diagnosis** | -0.41 | 0.41 | 1.03 | 0.31 |
|  | **Modified DAS** | 0.04 | 0.12 | 0.09 | 0.77 |
|  | **PGA** | -0.08 | 0.21 | 0.13 | 0.72 |
|  | **CMAS** | -0.11 | 0.04 | 6.94 | 0.01 |
| **5** | **Intercept** | 6.50 | 1.88 | 12.00 | 0.00 |
|  | **Time since diagnosis** | -0.39 | 0.45 | 0.74 | 0.39 |
|  | **Modified DAS** | 0.03 | 0.13 | 0.04 | 0.83 |
|  | **PGA** | -0.20 | 0.20 | 0.97 | 0.33 |
|  | **CMAS** | -0.14 | 0.04 | 11.62 | 0.00 |

CMAS, Childhood Myositis Assessment Scale; DAS, Disease Activity Score; PGA, physician’s global assessment of disease activity

**Supplementary Table 5. Autoantibody features of patients treated with CYC compared to patients not treated with CYC^a^**

|  | **Treated with CYC (n=56)** | **Not treated with CYC (n=144)** | **Statistic** | **P-value** |
| --- | --- | --- | --- | --- |
| **Patients with an autoantibody result** | 51 (91.1%) | 122 (84.7%) |  |  |
| **Myositis-specific autoantibody^c^** | 28 (54.9%) | 62 (50.8%) |  |  |
| Anti-TIF1γ | 12 (23.5%) | 18 (14.8%) |  | 0.66^b^ |
| Anti-NXP-2 | 9 (17.5%) | 23 (18.9%) |  |  |
| Anti-MDA5 | 4 (7.8%) | 9 (7.4%) |  |  |
| Anti-Mi-2 | 2 (3.9%) | 7 (5.7%) |  |  |
| Anti-PL-7 | 1 (2.0%) | 0 (0.0%) |  |  |
| Anti-Jo-1 | 0 (0.0%) | 1 (0.8%) |  |  |
| Anti-SRP | 0 (0.0%) | 3 (2.5%) |  |  |
| Anti-SAE | 0 (0.0%) | 1 (0.8%) |  |  |
| **Myositis-associated autoantibodies** | 3 (5.8%) | 10 (8.2%) |  |  |
| Anti-PM-Scl | 2 (3.9%) | 5 (4.1%) |  | 0.24^b^ |
| Anti-Topo | 1 (2.0%) | 0 (0.0%) |  |  |
| Anti-U1RNP | 0 (0.0%) | 4 (3.3%) |  |  |
| Anti-U3RNP | 0 (0.0%) | 1 (0.8%) |  |  |
| **Unidentified autoantibodies** | 8 (15.7%) | 22 (18.0%) |  |  |
| **No detectable autoantibodies** | 12 (23.5%) | 28 (23.0%) |  |  |

^a^Data are presented as number (percentage)

^b^Analysis using Chi-square test

^c^Autoantibody data are presented as number and percentage of total patients with an autoantibody result within either the CYC or the non-CYC groups. Statistical analysis of the distribution of myositis-specific autoantibodies and myositis-associated autoantibodies across the CYC and non-CYC patient groups was performed using Fisher’s exact test, with separate analyses performed for myositis-specific autoantibodies and myositis-associated autoantibodies. Three patients in whom more than one autoantibody was detected were excluded from this analysis (1 CYC patient with U1RNP & TIF1γ, 1 non-CYC patient with PL-12 & Ro52, 1 non-CYC patient with Mi2 & NXP2).

**Supplementary Figure 1. Weighted and unweighted clinical measures over time for CYC and non-CYC treatment groups.** Mean **(A)** weighted modified DAS, **(B)** unweighted modified DAS, **(C)** weighted PGA, **(D)** unweighted PGA, **(E)** weighted CMAS and **(F)** unweighted CMAS were plotted over time. Where the confidence intervals for these estimates overlap in the weighted datasets, balance has been achieved between the CYC and non-CYC treatment groups.


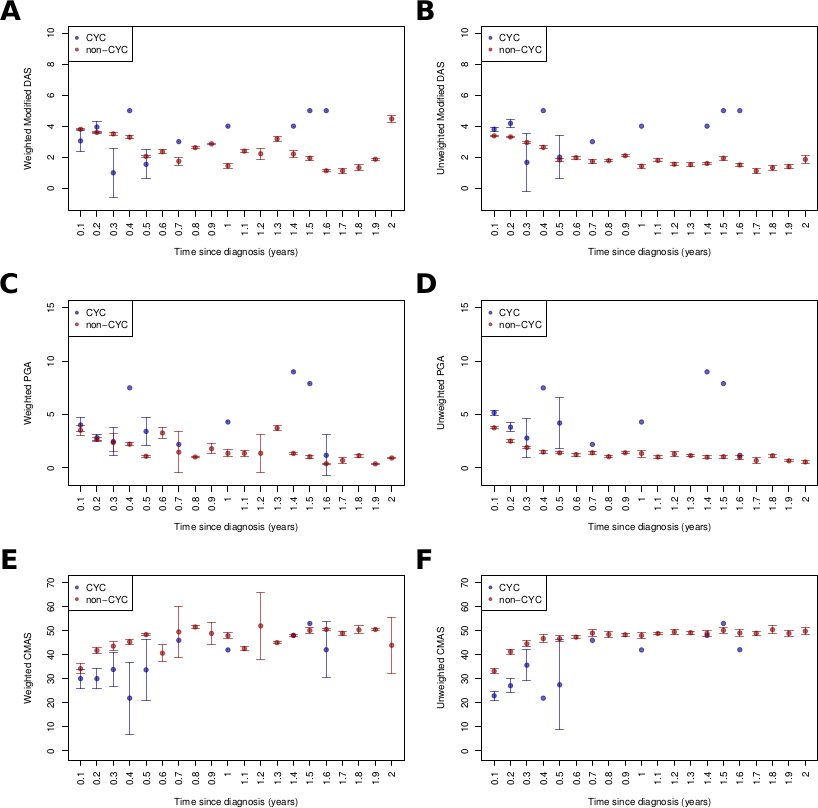


**REFERENCES FOR SUPPLEMENTARY MATERIAL**

1 Team RDC. R : A Language and Environment for Statistical Computing. Vienna Austria R Found. Stat. Comput. 2015;:ISBN 3-900051-07-0. doi:10.1111/j.1365-2621.1979.tb03829.x

2 Lam CG, Manlhiot C, Pullenayegum EM, *et al.* Efficacy of intravenous Ig therapy in juvenile dermatomyositis. *Ann Rheum Dis* 2011;**70**:2089–94. doi:10.1136/ard.2011.153718

3 Zeger SL, Liang KY, Albert PS. Models for longitudinal data: a generalized estimating equation approach. *Biometrics* 1988;**44**:1049–60.http://www.ncbi.nlm.nih.gov/pubmed/3233245 (accessed 10 Mar2013).

4 Hojsgaard S, Halekoh U, Yan J. The R Package geepack for Generalized Estimating Equations. *J Stat Softw* 2006;**15**:1–11.

5 Talbot D, Atherton J, Rossi AM, *et al.* A cautionary note concerning the use of stabilized weights in marginal structural models. *Stat Med* 2015;**34**:812–23. doi:10.1002/sim.6378

6 Hernan MA, Robins JM. Estimating causal effects from epidemiological data. *J Epidemiol Community Heal* 2006;**60**:578–86. doi:10.1136/jech.2004.029496

7 Warnes GR, Bolker B, Bonebakker L, *et al.* gplots: Various R Programming Tools for Plotting Data. 2016.https://cran.r-project.org/web/packages/gplots/index.html (accessed 6 Dec2016).

8 Bohan A, Peter JB. Polymyositis and dermatomyositis (first of two parts). *N Engl J Med* 1975;**292**:344–7. doi:10.1056/NEJM197502132920706

9 Bohan A, Peter JB. Polymyositis and dermatomyositis (second of two parts). *N Engl J Med* 1975;**292**:403–7. doi:10.1056/NEJM197502202920807
